# Supplementary material for: Robust cullin-RING ligase function is established by a multiplicity of poly-ubiquitylation pathways
Source: eLife. 2019 Dec 23;8:e51163. doi: 10.7554/eLife.51163 (PMC6975927; doi:10.7554/eLife.51163)
Supplement: Table 3—source data 1. [file elife-51163-table3-data1.docx]

Table 3 - Source Data 1. P-values for the estimates of the rates of ubiquitin transfer to substrate *k_obs_* (sec^-1^)

|  | | | | Substrate prior to ubiquitin transfer* | | |
| --- | --- | --- | --- | --- | --- | --- |
| Substrate | Chain modifying E2 or E3 | E3 | S0 | | S1 | S2 |
| Cyclin E | Arih1 | SCF^Fbw7^ | 5.3 E-9 (*3.3 E-14*) | | 0.00018 (*3.7 E-7*) | 9.5 E-7 (*0.000026*) |
| β-Catenin | Arih1 | SCF^βTrCP^ | 5.6 E-8 (*4.5 E-12*) | | 0.0014 (*0.000012*) | 0.79 (*6.8 E-6*) |
| Cyclin E | Ube2D3 | SCF^Fbw7^ | 2.8 E-7 (*1.0 E-8*) | | 0.017 (*0.000099*) | 0.33 (0.53) |
| β-Catenin | Ube2D3 | SCF^βTrCP^ | 8.1 E-6 (*1.2 E-7*) | | 0.00018 (*0.000014*) | ND (*0.00063*) |
| Cyclin E | Ube2R2 | SCF^Fbw7^ | 3.4 E-7 (ND) | | 1.4 E-7 (ND) | 7.6 E-11 (ND) |
| β-Catenin | Ube2R2 | SCF^βTrCP^ | 2.1 E-9 (ND) | | 2.0 E-7 (ND) | 3.2 E-9 (ND) |
| Cyclin E | Arih1/Ube2R2 | SCF^Fbw7^ | 0.0023 (*3.6 E-11*) | | 0.16 (*1.9 E-9*) | 0.089 (*3.1 E-7*) |
| β-Catenin | Arih1/Ube2R2 | SCF^βTrCP^ | 0.000019 (*2.1 E-8*) | | 0.0021 (*0.000014*) | 0.000065 (*2.2 E-6*) |
| Cyclin E | Ube2D3/Ube2R2 | SCF^Fbw7^ | 4.5 E-12 (*3.1 E-11*) | | 9.1 E-6 (*5.8 E-6*) | 4.0 E-8 (*1.8 E-8*) |
| β-Catenin | Ube2D3/Ube2R2 | SCF^βTrCP^ | 3.5 E-6 (*8.4 E-8*) | | 0.000020 (*1.4 E-6*) | 1.6 E-6 (*1.8 E-6*) |
| Ub-Cyclin E | Ube2G1 | SCF^Fbw7^ | ND (ND) | | 3.7 E-7 (ND) | 1.4 E-7 (ND) |
