# Supplementary material for: Robust cullin-RING ligase function is established by a multiplicity of poly-ubiquitylation pathways
Source: eLife. 2019 Dec 23;8:e51163. doi: 10.7554/eLife.51163 (PMC6975927; doi:10.7554/eLife.51163)
Supplement: Supplementary file 1. [file elife-51163-supp1.docx]

**Template:**

| **Key Resources Table** | | | | |
| --- | --- | --- | --- | --- |
| **Reagent type (species) or resource** | **Designation** | **Source or reference** | **Identifiers** | **Additional information** |
| cell line (homo-sapiens) | HEK 293T/17 | ATCC | CRL-11268; RRID CVCL_1926 |  |
| cell line (*homo-sapiens)* | HeLa | ATCC | CCL-2; RRID CVCL_0030 |  |
| cell line (*homo-sapiens)* | MRC5 | ATCC | CCL-171; RRID CVCL_0440 |  |
| cell line (*homo-sapiens)* | 293T-FiTx | PMID:29103612 |  | Ray Deshaies lab |
| cell line (*homo-sapiens* | NALM-6 | PMID:29103612 |  | Mike Tyers lab |
| cell line (*homo-sapiens* | 293T-FiTx UBE2R1Δ | this paper |  | Kleiger lab on request |
| cell line (*homo-sapiens* | HEK 293T/17 UBE2R1/2Δ | this paper |  | Sicheri lab on request |
| cell line (*homo-sapiens* | NALM-6 UBE2R1Δ | this paper |  | Tyers lab on request |
| cell line (*homo-sapiens* | NALM-6 UBE2R1/2Δ | this paper |  | Tyers lab on request |
| recombinant DNA reagent | pET-11b human Ube2R2 | PMID:24129577 | GKD1 | Kleiger lab on request |
| recombinant DNA reagent | pAL Cul1-NTD | PMID:16275325 |  |  |
| recombinant DNA reagent | pGEX GST-thrombin-Rbx1-rbs-Cul1CTD | PMID:16275325 |  |  |
| recombinant DNA reagent | pGEX GST-thrombin-Rbx1-rbs-K720R-Cul1-CTD | this study | GKD271 | Kleiger lab on request |
| recombinant DNA reagent | pGEX-4T1 GST-TEV-Ube2D3 | this study | GKD158 | Kleiger lab on request |
| recombinant DNA reagent | pGEX-4T1 GST-TEV-Ube2G1 | this study | GKD442 | Kleiger lab on request |
| recombinant DNA reagent | pET-11b human His6-K0-ubiquitin | this study | GKD323 | Kleiger lab on request |
| recombinant DNA reagent | pET-11b human His8-NEDD8 | this study | GKD345 | Kleiger lab on request |
| recombinant DNA reagent | pGEX-4T1 GST-TEV-ARIH1 | PMID:27565346 |  |  |
| recombinant DNA reagent | pGEX-4T1 GST-TEV-UBE2L3 | PMID:27565346 |  |  |
| recombinant DNA reagent | pX330 | PMID:24157548 |  |  |
| recombinant DNA reagent | pSpCas9(BB)-2A-Puro (pX459) | PMID:29038160 |  |  |
| recombinant DNA reagent | pLX-sgRNA 2X BfuA1 | PMID:29038160 |  |  |
| recombinant DNA reagent | pLX-sgRNA 2X BsiW1 AAVS1 Hygro | PMID:29038160 |  |  |
| recombinant DNA reagent | pLX-sgRNA 2X BsiW1 AAVS1 Neo | PMID:29038160 |  |  |
| sequence-based reagent | *UBE2R1* guide sequence for pX330 | this study | guide RNA sequence | GGAGAGTATGGGTAGTCGAT |
| sequence-based reagent | CRISPR Validation *UBE2R1* | this study | 5' primer | GCAACCGGATCCAGGATACGGTGTCCCTCACA |
| sequence-based reagent | CRISPR Validation *UBE2R1* | this study | 3' primer | GCAACCCTCGAGCCGGAGGACGAGGACG |
| sequence-based reagent | Real Time PCR *UBE2R1* | this study | 5' primer | CCTGAGTGTGATCTCCCTCCT |
| sequence-based reagent | Real Time PCR *UBE2R1* | this study | 3' primer | TGTCTGTGTACTCCCGATCCT |
| sequence-based reagent | Real Time PCR *UBE2R2* | this study | 5' primer | ACTACCCCTATTCACCACCTAC |
| sequence-based reagent | Real Time PCR *UBE2R2* | this study | 3' primer | AGGCGGATGAAGAATCGAAATG |
| sequence-based reagent | Real Time PCR *GAPDH* | this study | 5' primer | GTGAACCATGAGAAGTATGACAAC |
| sequence-based reagent | Real Time PCR *GAPDH* | this study | 3' primer | CATGAGTCCTTCCACGATACC |
| sequence-based reagent | *UBE2R1* exon 2 HDR | this study | oligo | TCTTGGGGCTGGGCTGGACTGAGCCACCTGCCTTCTGCCCCTGC  AGGCGCGCCTCAAGTTATAATAATAATAAGTCCAAAAAAACCACAAGCCAGAAAGGGTTCCATGGACTCTCCACCAGCCTTTCGGTTCCTGACCAAGATGTGGCACCCTAACATCTACGAGGTGAG |
| sequence-based reagent | *UBE2R1* guide sequence #1 for pX459 | this study | guide RNA sequence | GGGCGATCTATACAACTGGG |
| sequence-based reagent | *UBE2R1* guide sequence #2 for pX459 | this study | guide RNA sequence | GCCCCCCAACACCTACTACG |
| sequence-based reagent | *UBE2R2* guide sequence #1 for pX459 | this study | guide RNA sequence | GTCCGACCTCTACAACTGGG |
| sequence-based reagent | *UBE2R2* guide sequence #2 for pX459 | this study | guide RNA sequence | GAAGTAGCCGCCTTCGTAG |
| sequence-based reagent | *UBE2R1* guide sequence #1 for pLX-sgRNA 2X BsiW1 Neo | this study | guide RNA sequence | GGGCGATCTATACAACTGGG |
| sequence-based reagent | *UBE2R1* guide sequence #2 for pLX-sgRNA 2X BsiW1 Neo | this study | guide RNA sequence | GTCAGGAACCGAAAGGCTGG |
| sequence-based reagent | *UBE2R2* guide sequence #1 for pLX-sgRNA 2X BsiW1 Hygro | this study | guide RNA sequence | GCGACCTCTACAACTGGGAGG |
| sequence-based reagent | *UBE2R2* guide sequence #2 for pLX-sgRNA 2X BsiW1 Hygro | this study | guide RNA sequence | GGTCGGACTCGTCCACC |
| sequence-based reagent | GFP-1 guide sequence for pLX-sgRNA 2X BsiW1 Hygro | this study | guide RNA sequence | GTGAACCGCATCGAGCTGAG |
| sequence-based reagent | *AAVS1* guide sequence for pLX-sgRNA 2X BsiW1 Neo | this study | guide RNA sequence | GGGGCCACTAGGGACAGGAT |
| sequence-based reagent | 5’ oligo for 1032 bp AAVS1 insert | this study | oligo | TTATATATCTTGTGGAAAGGACGAAACACCGTACGTGTGCC  ATCTCTCGTTTCTTA |
| sequence-based reagent | 3’ oligo for 1032 bp AAVS1 insert | this study | oligo | ATTTTAACTTGCTATTTCTAGCTCTAAAACGTACGCACAAAG  GGAGTTTTCCACA |
| sequence-based reagent | 5' Hygromycin oligo | this study | oligo | cccgcgcggtgttccgcattctgcaagcctCTGGCCCAACCATGAAAAA |
| sequence-based reagent | 3' Hygromycin oligo | this study | oligo | ctgccatttgtctcaagatctagaattCTCATTACTGCAGATCCTTT |
| sequence-based reagent | 5’ Neomycin oligo | this study | oligo | cccgcgcggtgttccgcattctgcaagcctATAATACGACAAGGTGAGGA |
| sequence-based reagent | 3’ Neomycin oligo | this study | oligo | actgccatttgtctcaagatctagaattAATTGATCCCCTCAGAAG |
| sequence-based reagent | *UBE2G1* guide sequence #1 for pLX-sgRNA 2X BfuA1 | this study | guide RNA sequence | GATGGTTTCCACAGTGTGGAT |
| sequence-based reagent | *UBE2G1* guide sequence #2 for pLX-sgRNA 2X BfuA1 | this study | guide RNA sequence | GCTGGCTCCCTATCCACACTG |
| sequence-based reagent | AZ-green guide sequence | this study | guide RNA sequence | GGCCACAACTTCGTGATCGA |
| sequence-based reagent | Internal primer for blasticidin resistance | this study | sequencing primer | TGGTTATGTGTGGGAGGG |
| sequence-based reagent | siRNA:control | Dharmacon | D 001810 10 05 | ON-TARGET |
| sequence-based reagent | siRNA:*UBE2G1* | Dharmacon | L 010154 00 0005 | ON-TARGET |
| antibody | anti-αTUBULIN DM1A (Mouse monoclonal) | Cell Signaling Technology | 3873; RRID AB_1904178 | WB: 1:5000 (all figures) |
| antibody | anti-CYCLIN E1 (HE12) (Mouse monoclonal) | Cell Signaling Technology | 4129; RRID AB_2071200 | WB: 1:2000 (all figures) |
| antibody | anti-ARIH1 (Goat polyclonal) | Abnova | PAB6322; RRID AB_1571353 | WB: 1:500 (Fig. 5a) |
| antibody | anti-β-CATENIN (Mouse monoclonal) | BD Bioscienes | 610153; RRID AB_397554 | WB: 1:2000 (Fig. 5a) |
| antibody | anti-IκBα (L35A5) (Mouse monoclonal) | Cell Signaling Technology | 4814; RRID AB_390781 | WB: 1:1000 (Fig. 5d; Fig 5 - supp. 3b) |
| antibody | anti-UBE2R1 (Rabbit monoclonal) | Abcam | ab204515 | WB: 1:5000 (Fig. 5 - supp 1a); IF: 1:50 (Fig. 5 - supp 5) |
| antibody | anti-UBE2R1 (H81) (Rabbit polyclonal) | Santa Cruz | sc5616; RRID AB_2276050 | WB: 1:1000 (all figures except the above) |
| antibody | anti-UBE2R2 (H51) (Rabbit polyclonal) | Santa Cruz | sc134628; RRID AB_2010705 | WB: 1:1000 (all figures) |
| antibody | anti-UBE2G1 (G832) (Mouse monclonal) | Santa Cruz | sc100619; RRID AB_1130981 | WB: 1:250 (all figures) |
| antibody | anti-p27 (Rabbit monoclonal) | Cell Signaling Technology | 3686; RRID AB_2077850 | WB: 1:2000 (Fig. 5 - supp 1a) |
| antibody | anti-p27 (Mouse monoclonal) | BD Biosciences | 610242; RRID AB_397637 | WB: 1:2000 (all figures except the above) |
| antibody | anti-Hif1α (Rabbit monoclonal) | Abcam | ab179483; RRID AB_2732807 | WB: 1:500 (Fig. 7a) |
| antibody | anti-UBE2D3 (Rabbit monoclonal) | Abcam | ab176568 | WB: 1:5000 (Fig. 5a) |
| antibody | Goat anti-Rabbit IgG, Alexa Fluor 488 | Thermo Fisher | A11004 | IF: 1:500 (Fig. 5 - supp 5) |
| antibody | Goat anti-Mouse IgG, Starbright Blue B700 | BioRad | 12004159 | WB: 1:3000 |
| antibody | Goat-anti-Rabbit IgG, Starbright Blue B700 | BioRad | 12004162 | WB: 1:3000 |
| peptide, recombinant protein | β-catenin peptide | New England Peptide | custom | N-Ac-KAWQQQSYLD(pS)GIH(pS)-GATTTAPRRASY |
| peptide, recombinant protein | Cyclin E peptide | New England Peptide | custom | N-Ac-KAMLSEQNRASPLPSGLL(pT)-PPQ(pS)GRRASYHHHHHH |
| peptide, recombinant protein | Human recombinant ubiquitin | Boston Biochem | U-100H |  |
| peptide, recombinant protein | TNFα | R&D | 210-TA-020 |  |
| commercial assay or kit | Lipofectamine 3000 | Invitrogen | L3000008 |  |
| commercial assay or kit | Lipofectamine RNAiMAX | Invitrogen | 13778030 |  |
| commercial assay or kit | SuperScript III First-Strand Synthesis System | Invitrogen | 18080051 |  |
| commercial assay or kit | Universal SYBR Green Supermix | BioRad | 1725270 |  |
| commercial assay or kit | RNeasy Mini Kit | Qiagen | 74104 |  |
| commercial assay or kit | Pierce BCA Assay Kit | Thermo Fisher | 23225 |  |
| commercial assay or kit | QuickExtract DNA Solution | Lucigen | QE09050 |  |
