# Supplementary material for: Robust cullin-RING ligase function is established by a multiplicity of poly-ubiquitylation pathways
Source: eLife. 2019 Dec 23;8:e51163. doi: 10.7554/eLife.51163 (PMC6975927; doi:10.7554/eLife.51163)
Supplement: Supplementary file 2. [file elife-51163-supp2.docx]

| Figure Number | Experiment | [Ub] (μM) | [E1] (μM) | [UBE2D3] (μM) | [UBE2R2] (μM) | [UBE2L3] (μM) | [ARIH] (μM) | [UBE2G1] (μM) | [SCF] (μM) | [substrate] (μM) |
| --- | --- | --- | --- | --- | --- | --- | --- | --- | --- | --- |
| 2b,e;S2-2a;S2-3a;S2-4a,c,e,g;S2-6a;S2-7a;S2-8a;S2-9a | QF, saturating | 40 | 0.25 | 10 | 10 | 10 | 2.5 | 12.5 | 0.5 | 0.1 |
| 2d,f;S2-2c;S2-3c;S2-5a,b;S2-6c;S2-7c;S2-8c;S2-9c | QF, 2x cellular | 40 | 0.25 | 3.7 | 0.5 | 3.7 | 0.36 | N/A | 0.5 | 0.1 |
| 1a-c;S1a-f;S7b,c | *K_m_* | 60 | 1 | titration | titration | 10 | titration | titration | 0.1 | 5 |
| 4;S4 | 1/4x through 4x cellular titration | 40 | 0.25 | titration | titration | 3.7 | titration | N/A | 0.5 | 0.1 |
| 7c | single-encounter | 40 | 0.25 | 3.7 |  | 3.7 | 0.36 | 13 | 0.5 | 0.1 |
| 7e | QF, saturating, UBE2G1 | 40 | 0.25 | N/A | N/A | N/A | N/A | 12.5 | 0.5 | 0.1 |

Supplementary File 2: Experimental Conditions for Ubiquitylation Reactions

QF, Quench Flow; N/A, not applicable; saturating is E2 or ARIH1 for the SCF-substrate complex; cellular is E2 or ARIH1 concentrations determined by SRM mass-spectrometry. All Quench Flow and single-encounter reactions contain a final unlabeled peptide concentration of 100 μM.
